# An Incidental Case of a Rare Ventricular Septal Defect (VSD): Does Infective Endocarditis Ger-Bode Well?

**DOI:** 10.7759/cureus.60677

**Published:** 2024-05-20

**Authors:** Marc T Zughaib, Jessica LaVoie, Naveen Multani, Saba Darda

**Affiliations:** 1 Cardiology, Ascension Providence Hospital, Southfield, USA; 2 Internal Medicine, Ascension Providence Hospital, Southfield, USA

**Keywords:** echocardiography, transesophageal echocardiography (tee), infective endocarditis, cardiology, ventricular septal defect (vsd), gerbode defect

## Abstract

The Gerbode defect is a rare ventricular septal defect (VSD) between the left ventricle (LV) and right atrium (RA). We describe a challenging case of a rare acquired Gerbode defect from infective endocarditis.

A 73-year-old male presented for left lower extremity edema and shortness of breath with exertion. He was discharged from the hospital one week prior after being diagnosed with right hip septic arthritis. A transthoracic echocardiogram (TTE) did not demonstrate an abscess or vegetation, but was significant for severely elevated tricuspid regurgitation velocity and pulmonary artery (PA) systolic pressure of 70 mm Hg without structural changes to the right ventricle or RA. A transesophageal echocardiogram (TEE) was performed due to these abnormal values and demonstrated a VSD between the LV and RA. This type of defect is known as a Gerbode defect, which is suggestive of an aortic root abscess. The patient ultimately was transferred to a tertiary care center, and the Gerbode defect with aortic root abscess was confirmed by direct visualization.

This case reports a unique case of an acquired Gerbode defect secondary to infective endocarditis. Our patient's defect was noted to be above the tricuspid valve, which essentially confirmed the etiology as a VSD. Although the TEE did not demonstrate a clear aortic root abscess, direct visualization during the surgical intervention confirmed this suspicion. Prompt diagnosis of the Gerbode defect allowed the patient to receive urgent surgical intervention.

Gerbode defects are rare but clinically important complications of infective endocarditis. This case highlights the importance of maintaining a high level of suspicion, especially if the values obtained during TTE do not fully explain a patient's clinical presentation. A high level of suspicion leading to a timely diagnosis of this condition is essential in preventing further valvular destruction and allowing prompt surgical intervention.

## Introduction

Previously, ventricular septal defects (VSDs) with communication between the left ventricle (LV) and right atrium (RA) were rare, but these defects have been increasingly reported in recent years [1-3]. Anatomically, VSDs are defined by their location relative to the tricuspid valve. Supravalvular defects, also known as direct defects, are superior to the septal leaflet of the tricuspid valve. Infravalvular or indirect defects are present inferior to the septal leaflet of the tricuspid valve and are the most common. Intermediate defects include portions of both the inferior and superior portions of the septum in relation to the tricuspid valve and are the least common presentation [1].

The Gerbode defect is a rare VSD between the LV and RA that can be present congenitally but has increasingly been reported from acquired causes [4]. Acquired causes include procedures such as native valve replacement, atrioventricular (AV) node ablation, tricuspid annuloplasty, or cardiac biopsy. Non-iatrogenic causes include infective endocarditis with or without perivalvular abscess, myocardial infarction, and trauma. 

We describe a challenging case of a rare acquired Gerbode defect from infective endocarditis.

## Case presentation

A 73-year-old male presented for left lower extremity edema and shortness of breath with exertion. He was discharged from the hospital one week prior after being diagnosed with right hip septic arthritis. His septic joint was secondary to methicillin-resistant *Staphylococcus epidermidis* (MRSE) confirmed on blood cultures. Upon physical examination, a grade IV/VI systolic murmur at the left lower sternal border and bilateral 2+ lower extremity edema were present. The patient was admitted with consultation to the infectious disease and orthopedic surgery teams. Cardiology was also consulted to rule out infective endocarditis due to the positive blood cultures and the new murmur on examination. 

**Figure 1:**
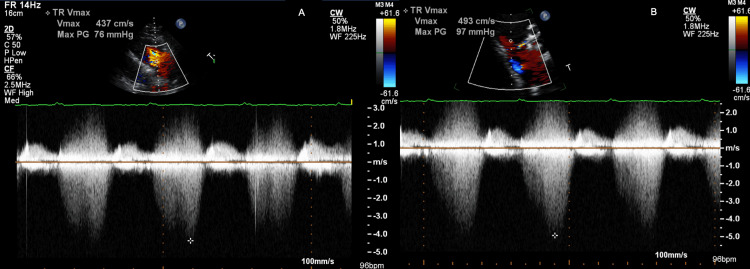
TTE image of RV inflow view. Panel A with a Doppler waveform across the tricuspid valve with a Vmax of 4.37 m/s originally described as "tricuspid regurgitation." Evidence of a double envelope across the tricuspid valve secondary to the VSD seen at the white star. Panel B with a double dagger envelope, Vmax of 4.93 m/s, occurring in mid-systole suggestive of a VSD. TTE: transthoracic echocardiogram; RV: right ventricle; VSD: ventricular septal defect

The patient's white blood count ranged from 9000 to 16,000 throughout the hospitalization with positive blood cultures for MRSE on seven separate occasions. Initial electrocardiogram (ECG) demonstrated sinus tachycardia, first-degree AV block, and QRS duration of 88 ms. Transthoracic echocardiogram (TTE) demonstrated an ejection fraction of 55-60% with no evidence of vegetation. There were moderate tricuspid regurgitation (TR) and severe pulmonary hypertension with a pulmonary artery (PA) systolic pressure of 70 mm Hg. There was no evidence of right ventricular (RV) or RA enlargement. Additionally, the TR Doppler profile demonstrated a "double dagger" appearance that occurred during mid-systole (Figure 1). 

Due to the abnormal TR velocities, abnormal double dagger Doppler signal, and normal size of RV and RA, a closer review of the TTE raised suspicion for a left to right shunt from the LV to the RA. Further evaluation of the abnormal TR values was pursued with a transesophageal echocardiogram (TEE). 

**Figure 2:**
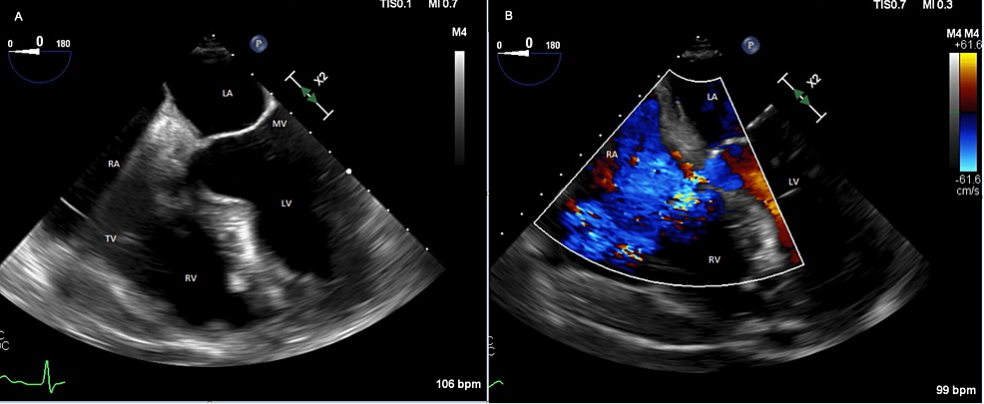
Transesophageal view of the Gerbode defect. Panel A is a four-chamber view with the noted Gerbode defect superior to the tricuspid valve. Panel B with Doppler color wave through the Gerbode defect, from the left ventricle into the right atrium.

During the TEE, the previously described TR envelope was now seen by sampling above the tricuspid valve. This regurgitant jet originated from the LV and entered into the RA (Figure 2). There was mild TR noted, which allowed for the confirmation that the previously noted TR on the TTE was actually regurgitation from a VSD from the LV to the RA known as a Gerbode defect. There was no evidence of vegetation or aortic root abscess appreciable in the imaging study. 

After diagnosing the Gerbode defect, the patient was ultimately transferred to a tertiary care center capable of surgical repair. Despite no clear aortic root abscess on TEE, a Gerbode defect is pathognomonic for an abscess. During surgical intervention, the Gerbode defect and aortic root abscess were confirmed by direct visualization. The patient underwent successful debridement of aortic root abscess, autologous pericardial patch placement, aortic valve replacement, and tricuspid valve annuloplasty. He continued to receive IV antibiotics throughout the periprocedural timeframe as well. He ultimately did well throughout the post-procedural timeframe and was discharged home. 

## Discussion

This case reports a unique case of an acquired Gerbode defect secondary to infective endocarditis. Endocarditis has been shown to cause a shunt between the LV to the RA by reopening a prior congenital defect, by widening a small shunt, or by destructive perforation of the septum [4]. Gerbode defects are typically described as a congenital defect. However, due to the increased occurrence of invasive cardiovascular procedures and improved cardiac diagnostic techniques, the number of acquired cases of Gerbode defect has increased [5]. Infective endocarditis-associated Gerbode defects have more than doubled over the past 20 years. From 1994 to 2004, there were eight reported cases which increased to 20 new cases reported from 2005 to 2014 [6,7]. 

Gerbode defects have been reported secondary to endocarditis from *S. aureus*, *S. haemolyticus*, *S. mutans*, *Haemophilus aphrophilus*, *Cardiobacterium hominis*, *S. pneumoniae*, and *S. lugdunensis* [7]. While there are rare case reports of *S. epidermidis* endocarditis leading to aortic valve abscess, there are no cases of *S. epidermidis* leading to Gerbode defect of native tricuspid and native tricuspid aortic. *S. epidermidis* is one of the most common causes of prosthetic valve endocarditis with approximately 40% of cases being linked to coagulase-negative staphylococci (CoNS) [8], whereas CoNS only account for approximately 5% of native valve endocarditis [9].

Our patient's defect was noted to be above the tricuspid valve, which essentially confirmed the etiology as a VSD. Although the TEE did not demonstrate a clear aortic root abscess, direct visualization during the surgical intervention confirmed this suspicion. Prompt diagnosis of the Gerbode defect allowed the patient to receive urgent surgical intervention. This case highlights the importance of maintaining a high level of suspicion, especially if the values obtained during TTE do not fully explain a patient's clinical presentation.

## Conclusions

Gerbode defects are rare but clinically important complications of infective endocarditis. A high level of suspicion leading to a timely diagnosis of this condition is essential in preventing further valvular destruction and allowing prompt surgical intervention. 
